# Lymphocytes in tumor-draining lymph nodes co-cultured with autologous tumor cells for adoptive cell therapy

**DOI:** 10.1186/s12967-022-03444-1

**Published:** 2022-05-23

**Authors:** Kazumi Okamura, Satoshi Nagayama, Tomohiro Tate, Hiu Ting Chan, Kazuma Kiyotani, Yusuke Nakamura

**Affiliations:** 1 Cancer Precision Medicine Center, Japanese Foundation for Cancer Research, 3-8-31 Ariake, Koto-ku, Tokyo, 135-8550 Japan; 2 Department of Gastroenterological and Surgery, Cancer Institute Hospital, Japanese Foundation for Cancer Research, Tokyo, Japan

**Keywords:** Colorectal cancer, Adoptive T cell therapy, Tumor-draining lymph nodes, Tumor-infiltrating lymphocytes

## Abstract

**Background:**

Tumor-draining lymph nodes (TDLNs) are primary sites, where anti-tumor lymphocytes are primed to tumor-specific antigens and play pivotal roles in immune responses against tumors. Although adoptive cell therapy (ACT) using lymphocytes isolated from TDLNs were reported, characterization of immune activity of lymphocytes in TDLNs to tumor cells was not comprehensively performed. Here, we demonstrate TDLNs to have very high potential as cell sources for immunotherapy.

**Methods:**

Lymphocytes from TDLNs resected during surgical operation were cultured with autologous-tumor cells for 2 weeks and evaluated tumor-reactivity by IFNγ ELISPOT assay. We investigated the commonality of T cell receptor (TCR) clonotypes expanded by the co-culture with tumor cells with those of tumor infiltrating lymphocytes (TILs).

**Results:**

We found that that TCR clonotypes of PD-1-expressing CD8^+^ T cells in lymph nodes commonly shared with those of TILs in primary tumors and lymphocytes having tumor-reactivity and TCR clonotypes shared with TILs could be induced from non-metastatic lymph nodes when they were co-cultured with autologous tumor cells.

**Conclusion:**

Our results imply that tumor-reactive effector T cells were present even in pathologically non-metastatic lymph nodes and could be expanded in vitro in the presence of autologous tumor cells and possibly be applied for ACT.

**Supplementary Information:**

The online version contains supplementary material available at 10.1186/s12967-022-03444-1.

## Background

Colorectal cancer (CRC) shows high incidence worldwide and is the most common cause of cancer-related death among Japanese women [1]. Although a 5-year survival rate of all colorectal cancer patients is as good as at approximately 70%, the survival rate deteriorated steeply to ~ 20% among those with distant metastasis. Therefore, there is an urgent need to develop new treatment modalities to improve the prognosis of colorectal cancer patients at advanced stages. At present, treatment options for CRC are mainly based on the stages; CRC patients at early stages usually receive surgery followed by chemotherapy when their stage is III (or IIb) and those patients who are at advanced stages receive surgery when tumors are resectable or often receive a combination of chemotherapy and radiotherapy as neoadjuvant treatment before the surgery [1, 2]. Recently, immunotherapy, particularly immune checkpoint inhibitors, is established as one of the treatment options, but its efficacy is limited to a small subset of CRC patients, whose tumors have microsatellite instability (MSI) associated with deficiency in mismatch repair (MMR) genes [3, 4]. Other types of immunotherapies include cancer-specific vaccines (peptide vaccines, dendritic cell vaccines, etc.) and ACT [5, 6]. One of the established ACTs include the use of TILs, which could be activated and expanded in vitro, and subsequently administered to patients. TIL therapy has shown clinical benefits for some cancer types, particularly for melanoma [7–10] and renal cancer [11], but it is not so effective for other cancer types [5, 12].

Alternatively, engineered CAR (chimeric antigen receptor) T cells, which have chimeric gene consisting a single chain variable fragment obtained from antibody against cell-surface molecules and T cells signaling domain (chimeric antigen receptor) such as anti-CD19 antibody for B-cell lymphoma or leukemia, have been shown clinically very effective. On the other hand, severe side effects were reported in patients who were treated with Her2-specific or CEA-specific CAR-T cell therapies probably by the on-target off-tumor effect because these molecules are expressed at low levels in normal cells [13–15]. Therefore, a novel safer and effective approach that cover a wider patient population should be developed.

During surgical operation of colorectal cancer, a tumor tissue(s) is excised together with their TDLNs that contain tumor-specific T cells. These TDLNs are important as primary sites for the host anti-tumor immune responses [16–19]. Moreover, it was reported that TDLNs with metastasis contained lymphocytes sharing common T cell receptor clonotypes with primary tumor tissues [20].Cell therapy with lymphocytes prepared from sentinel lymph nodes was also reported [21]. TDLNs may contain tumor-reactive T cells possibly at high levels and lymphocytes in TDLNs might be less exhausted rather than TILs. Hence, in this study, we investigated a possibility to utilize non-metastatic lymph nodes as cell sources to obtain tumor-reactive T cells. We here report that tumor-reactive T cells in non-metastatic lymph nodes could be expanded in vitro and suggest a new therapeutic approach for adoptive cell therapy.

## Materials and methods

### Patients

Patients with colorectal cancer who received surgical procedure were enrolled. Written informed consent was obtained before their surgery. Tumor staging was judged according to the Japanese Classification of Colorectal Carcinoma, 8th Edition [1]. Parts of a primary tumor and multiple lymph nodes were collected from individual patients. The study protocol was approved by the Institutional Review Board of Cancer Institute Hospital of Japanese Foundation for Cancer Research (2018-1021).

### Establishment of patient-derived tumor cell lines

Tumor tissue was mechanically dissociated into small tumor pieces using gentleMACS™ Dissociator (Miltenyi Biotec) and treated with 100 IU/ml collagenase (Thermo Fisher Scientific) and 50 ng/ml DNase I (Veritas) for 60 min at 37 ℃. After washing with PBS containing antibiotics (1 × penicillin and streptomycin (Wako)) and 0.5% BSA, the cell pellets were cultured in collagen-coated plates with CRC medium. CRC medium was composed of StemPro hESC SFM medium (Gibco) supplemented with 0.1 mM 2-Mercaptoethanol, 10 μM Y-27632 (LC Laboratories), 8 ng/ml bEGF (Peprotech) and Penicillin–Streptomycin-Amphotericin B (Wako). Tumor cell lines were cultured at 37 ℃ at 5% CO_2_ and tumor cell lines with less than 10 passages were used for experiments [22].

### Separation and culture of lymphocytes from lymph nodes

Lymph nodes were surgically resected and divided into two pieces. Half of them were used for pathological evaluation and half of the remaining ones was cut into 2–3 mm^3^ pieces and each piece was placed in a 24-well plate in 2 ml of lymphocyte medium and high-dose recombinant human IL-2 (6000 IU/ml) (R&D Systems, Minneapolis, USA). Lymphocyte medium was composed of RPMI-1640 (Wako)/AIM V (Gibco) supplemented with 12.5 mM HEPES, 2-Mercapto-ethanol and 5% human AB serum. Individual fragments were cultured at 37 ℃ at 5% CO_2_ for 2 to 3 weeks. Half of the culture media was replaced every 2 to 3 days. When lymphocytes exceeded 1 × 10^6^/ml or were nearly confluent, the cells were split [9, 23].

### Immunological evaluation of lymphocytes

An enzyme-linked immunospot (ELISPOT) assay kit of IFNγ (Mabtech) and that of Perforin (ImmunoSpot) were used to evaluate the tumor-specific T cells. Lymphocytes (1 × 10^5^/well) were seeded in 96-well plates and incubated with autologous tumor cells (1 × 10^5^/well), or anti-CD3 antibody (1 μg/ml) (for IFNγ ELISPOT assay) or PMA (50 ng/ml) /Ionomycin (1 μg/ml) (for Perforin ELISPOT assay). After 24 h of incubation, the assay was developed according to the standard protocol. The membranes were air-dried and the spots were evaluated using the ImmunoSpot plate reader using its associated software (Cellular Technologies Ltd.).

### Lymphocyte culture with tumor cells

One day before co-culture, lymphocytes were thawed and cultured in lymphocyte medium supplemented with 200 IU/ml IL-2 overnight at 37℃. Lymphocyte medium consisted of RPMI-1640 (Wako)/AIM V(Gibco) supplemented with 12.5 mM HEPES, 2-Mercapto-ethanol and 5% human AB serum. Tumor cells were stimulated overnight with 200 IU/ml of human recombinant IFNγ to facilitate HLA expression. 96-well U-bottom plates were coated with 5 μg/ml anti-CD28 (clone CD28.2) and kept overnight at 4 ℃. On the next day, tumor cells were dissociated to single cells with TrypLE (Gibco) and resuspended in lymphocytes medium. Lymphocytes were seeded at a density of 10^5^ cells/well (total 1 × 10^6^ cells) and co-cultured with tumor cells at a 20:1 ratio (lymphocytes: cancer cells, respectively). Co-culture was kept in the presence of 200 IU/ml IL-2 and 10 μg/ml anti-PD-1 antibody (clone EH12.2H7, Biolegend). Half of the medium was replaced two to three times per week. 1 week after the co-culture, lymphocytes were collected, counted, and replated at the concentration of 10^5^ cells/well with fresh tumor cells [24].

### Flow cytometric analysis

Fluorochrome-conjugated monoclonal antibodies specific to human TCRαβ (IP26), CD3 (HIT3a), CD4 (RPA-T4), CD8α (RPA-T8), CD19 (HIB19), CD56 (HCD56), PD-1(EH12.2H.7), TIM-3 (F38-2E2), CTLA4 (BNI3), LAG3 (7H2C65) and HLA class II (Tü39) were purchased from Biolegend. All samples were resuspended in PBS staining buffer containing 0.5% BSA, preincubated for 4 ℃ with FcR blocking reagent (Miltenyi Biotec), and then washed and stained with each of specific mAbs for 20 min at 4 ℃. Data were collected on a FACS verse (BD Biosciences) and analyzed using FlowJo (Tree Star) software.

### Transcriptome analysis

Libraries were prepared with the TrueSeq RNA Exome kit (Illumina, San Diego, USA) and sequenced on Nova Sex 6000 with 150 cycles of a pair-end sequencing module using the Illumina NovaSeq 6000 SP Reagent kit v1.5 (300 cycles). For obtaining expression profiles, the Subio Platform programs ver. 1.24 (Subio Inc., Amami, Japan) were used. We analyzed RNA-sequencing data by R packages (edgeR and limma) and use edgeR packaging to import, followed by the limma package with voom method, linear modeling and empirical Bayes moderation to assess differential expression and perform gene set testing by GSEA with the following statistical cutoffs: q-value less than 0.05; log_2_ fold change greater than 1 [25, 26].

### T cell receptor sequencing analysis

TCR sequencing was performed using the methods described previously [20, 27, 28]. In brief, we extracted total RNAs from 2 × 10^5^ T cells or tumor tissues, and cDNA was synthesized from total RNA with a 5’-Race adapter using the SMART library construction kit (Clontech). The TCRα and TCRβ cDNAs were amplified by PCR using a forward primer for the SMART adapter and a reverse primer corresponding to the constant region of TCRα and TCRβ. After adding the Illumina index sequences with barcode using the Nextra XT Index kit (Illumina), the prepared libraries were sequenced by 300-bp paired-end reads on the Illumina MiSeq platform, using MiSeq Reagent v3 600-cycles kit (Illumina). Obtained sequences were analyzed using Tcrip software.

### mRNA expression analysis

Transcripts were quantified on QuantStudio™ 5 Real-Time PCR System using TaqMan gene expression assays according to the manufacturer’s instructions (Applied Biosystems). Probes used were: TBX21; Hs00203436_m1, GATA3; Hs00231122_m1, ACTB; Hs01060665_g1. mRNA levels were normalized to ACTB level. Mean cycle threshold values (n = 2) were determined from amplification plots using QuantStudio Design and Analysis Software (Applied Biosystems).

### Genomic profiling of tumor tissue C215

Genomic DNAs (gDNAs) were extracted from sigmoid and rectal tumor tissues, and the corresponding adjacent normal tissues of C215 using AllPrep DNA/RNA Mini Kit (Qiagen). The extracted DNA was mechanically sheared (Covaris) to make to the average fragment size of 150 bp. Subsequently, library for comprehensive genomic profiling was constructed using Ion AmpliSeq™ Comprehensive Cancer Panel following the manufacturer’s protocol (Life Technologies) with an input of 40 ng DNA. The Ion AmpliSeq™ Comprehensive Cancer Panel consists of 16,000 primer pairs in four pools that covered all exons of 409 cancer-related genes [29]. The BAM files were analyzed using workflow AmpliSeq CCP w1.2-Tumor-Normal pair available in the Ion Reporter software (ver 5.10.3).

### Droplet digital PCR (ddPCR) of KRAS G12/G13 C177

To evaluate the mutation status of *KRAS* on codons G12/G13, ddPCR was performed using gDNA from tumor tissues of C177 and its adjacent four lymph nodes. This assay utilized ddPCR™ *KRAS* Screening Multiplex Kit (#1,863,506, BioRad) as described previously [30].

### Statistical analysis

Normally distributed data were compared using a two-sided unpaired student t-test. If the data did not meet the criteria of normality, Mann–Whitney U test was performed. Data are presented as mean ± standard error of the mean (SEM).

## Results

### In vitro enrichment of tumor-reactive T cells from non-metastatic lymph nodes

**Fig. 1:**
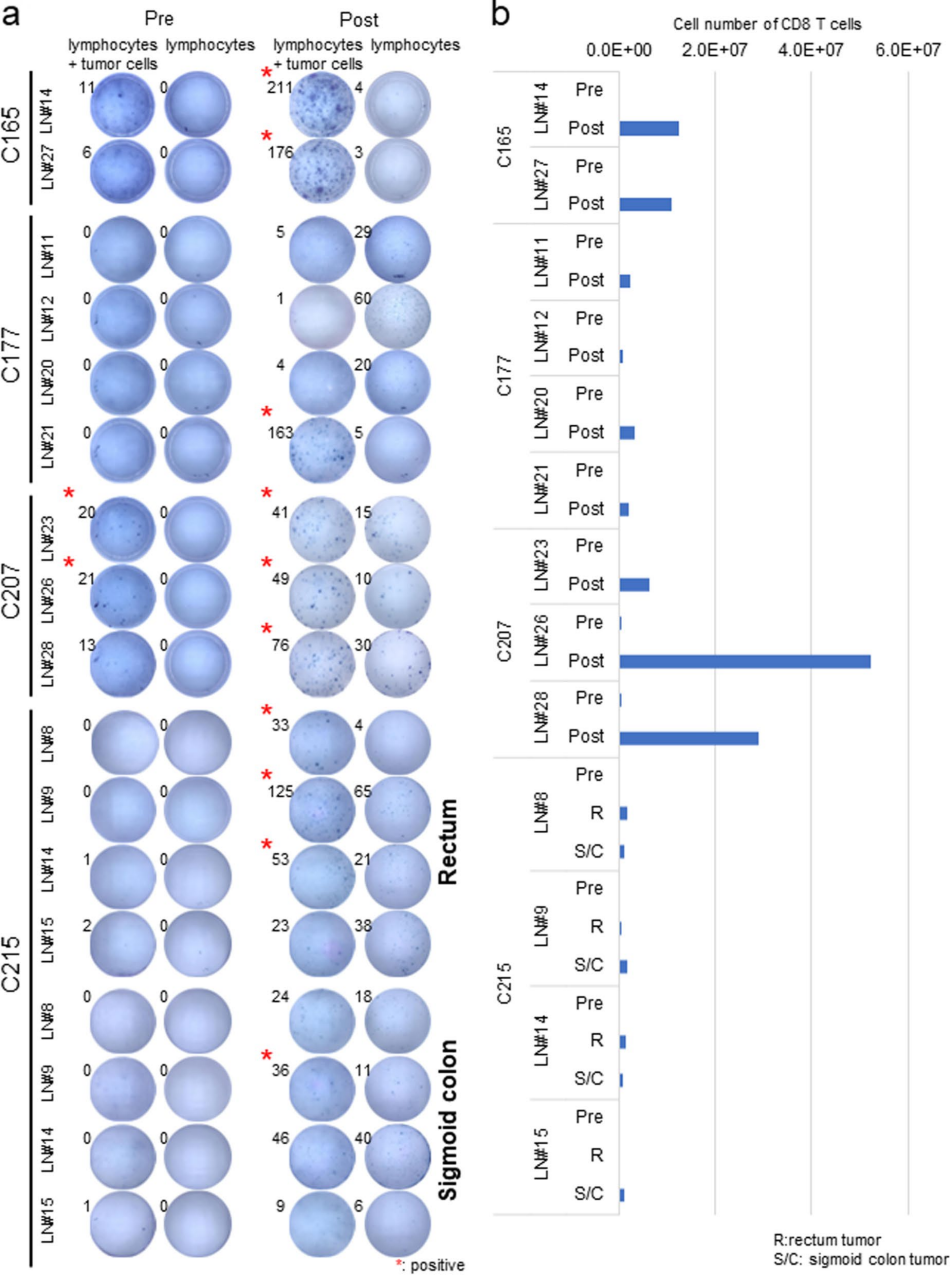
Screening and expansion of tumor-reactive T cells in non-metastatic lymph nodes. **a** Lymphocytes (1 × 10^5^/well) after making cell suspension were stimulated by autologous tumor cells (1 × 10^5^/well) and IFNγ secretions were detected by ELISPOT assay as shown Pre. Lymphocytes co-culture with autologous tumor-cells for 2 weeks were rested without tumor-cells for 2 days. Subsequently, lymphocytes (1 × 10^5^/well) were stimulated by autologous tumor-cells (1 × 10^5^/well) and IFNγ secretions were detected by ELISPOT assay as shown Post. Samples of which spot number (lymphocytes + tumor cells) is over 15 and more 1.5 folds than the negative control (only lymphocytes) were considered as positive. Experiments were conducted in duplicate. **b** Absolute live cell numbers were counted before and 2 weeks after co-culture with autologous tumor-cells. A part of lymphocytes was stained with anti-CD3, CD4, CD8α, CD56 and CD19 antibody and their expressions were evaluated by flow cytometry. The number indicates CD8^+^ T cells after co-culture with autologous tumor cells from 1 × 10^6^ total lymphocytes

To evaluate the tumor-reactive T cells in lymph nodes, autologous tumor-cell lines were established from colorectal tumor tissues of 7 CRC patients and their corresponding TDLNs as summarized in Table 1. For 3 CRC cases (C126, C149 and C152), we cut lymph nodes from both non-metastatic lymph nodes and metastatic lymph nodes into small pieces and cultured lymphocytes as similar to culturing TILs from tumor tissues [23]. After stimulation of lymphocytes with autologous tumor cells, we identified tumor-reactive T cells in lymphocytes isolated from non-metastatic lymph nodes of C126 by IFNγ ELISPOT assay, but we found tumor-reactive T cells in lymphocytes isolated from metastatic lymph nodes in two remaining cases (Additional file 1: Fig. S1a and Table 2). For additional 4 CRC cases (C165, C177, C207 and C215), we stimulated lymphocytes of non-metastatic lymph nodes by autologous tumor cells immediately after making cell suspension and then evaluated tumor-reactive T cells by IFNγ ELISPOT assay. In the case of C215 who possessed two tumors, one in the sigmoid colon and the other in the rectum, were conformed genetically to be independent by somatic mutation analysis (Additional file 5: Table S1). In this case, we stimulated lymphocytes from individual lymph nodes by either rectal and sigmoid colon tumor cells separately. Although we detected tumor-reactive T cells even in pre-stimulated lymphocytes of C207, we could detect no tumor reactive-T cells in other cases as shown “Pre” in Fig. 1a. Since a C207 patient was diagnosed to have Lynch syndrome, tumor-reactive T cells might be activated and expanded in vivo as highly immunogenic tumor.

**Table 1:** Characteristics of Colorectal cancer patients

Patient ID	Label	Lymph node No	Metastasis	Cell number (× 10^7^)	Primary site	Stage
C126	LN#1	241	−		Sigmoid colon	Stage III (T4aN1bM0)
	LN#2	241	−			
C149	LN#1	203	+		Cecum	Stage IVc (T4aN2bM1c)
	LN#3	202	+			
	LN#4	202	+			
	LN#5	202	+			
	LN#14	221	−			
C152	LN#15	241	−		Sigmoid colon	Stage IVa (T3N2bM1a)
	LN#22	241	+			
C165	LN#14	251	−	4.5	Rectum (Ra)	Stage IIa (T3N0M0)
	LN#27	251	−	4.2		
C177	LN#11	202	−	5.6	Ascending colon	Stage II (T4aN0M0)
	LN#12	202	−	11.0		
	LN#20	201	−	2.6		
	LN#21	201	−	2.4		
C207	LN#23	222	−	1.6	Transverse colon	Stage I (T2N0M0)
	LN#26	221	−	0.71		
	LN#28	221	−	1.3		
C215	LN#8	242	−	8.2	Sigmoid colon Rectum (Rb)	Stage IVa (T4bN1M1a)
	LN#9	242	−	11.0		
	LN#14	251	−	9.9		
	LN#15	251	−	10.0

**Table 2:** Summary of tumor-reactivity pre and post co-culture with autologous tumor cells

Patient ID	Primary site	Label	Regional or Non-regional	Metastasis	Tumor reactivity pre post	
C126	Sigmoid colon	LN#1	Regional	−	+	
		LN#2	Regional	−	+	
C149	Cecum	LN#1	Regional	+	+	
		LN#3	Regional	+	+	
		LN#4	Regional	+	+	
		LN#5	Regional	+	+	
		LN#14	Non-regional	−	−	
C152	Sigmoid colon	LN#15	Regional	−	−	
		LN#22	Regional	+	+	
C165	Rectum (Ra)	LN#14	Regional	−	−	+
		LN#27	Regional	−	−	+
C177	Ascending colon	LN#11	Regional	−	−	−
		LN#12	Regional	−	−	−
		LN#20	Regional	−	−	−
		LN#21	Regional	−	−	+
C207	Transverse colon	LN#23	Regional	−	+	+
		LN#26	Regional	−	+	+
		LN#28	Regional	−	−	+
C215	Rectum (Rb)	LN#8	Non-regional	−	−	+
		LN#9	Non-regional	−	−	+
		LN#14	Regional	−	−	+
		LN#15	Regional	−	−	−
C215	Sigmoid colon	LN#8	Regional	−	−	−
		LN#9	Regional	−	−	+
		LN#14	Non-regional	−	−	−
		LN#15	Non-regional	−	−	−

To further investigate the presence or absence of tumor-reactive T cells in non-metastatic lymph nodes, we attempted to enrich tumor-reactive T cells in vitro by the co-culture with autologous tumor cells for 2 weeks (Additional file 1: Fig. S1b). Interestingly, after this culture condition, we detected positive tumor-reactivities in lymphocytes by IFNγ ELISPOT assays as shown “Post” in Fig. 1a and Additional file 2: Figure S2a and CD8^+^ T cells were dominantly expanded in these four cases except the case C215 in which CD4^+^ T cells were dominantly expanded in all four lymph nodes (Fig. 1b), indicating that the co-culture with autologous tumor cells might be essential to expand rare tumor-reacting T cells from non-metastatic lymph nodes.

### Factors affecting differences in anti-tumor reactivity

**Fig. 2:**
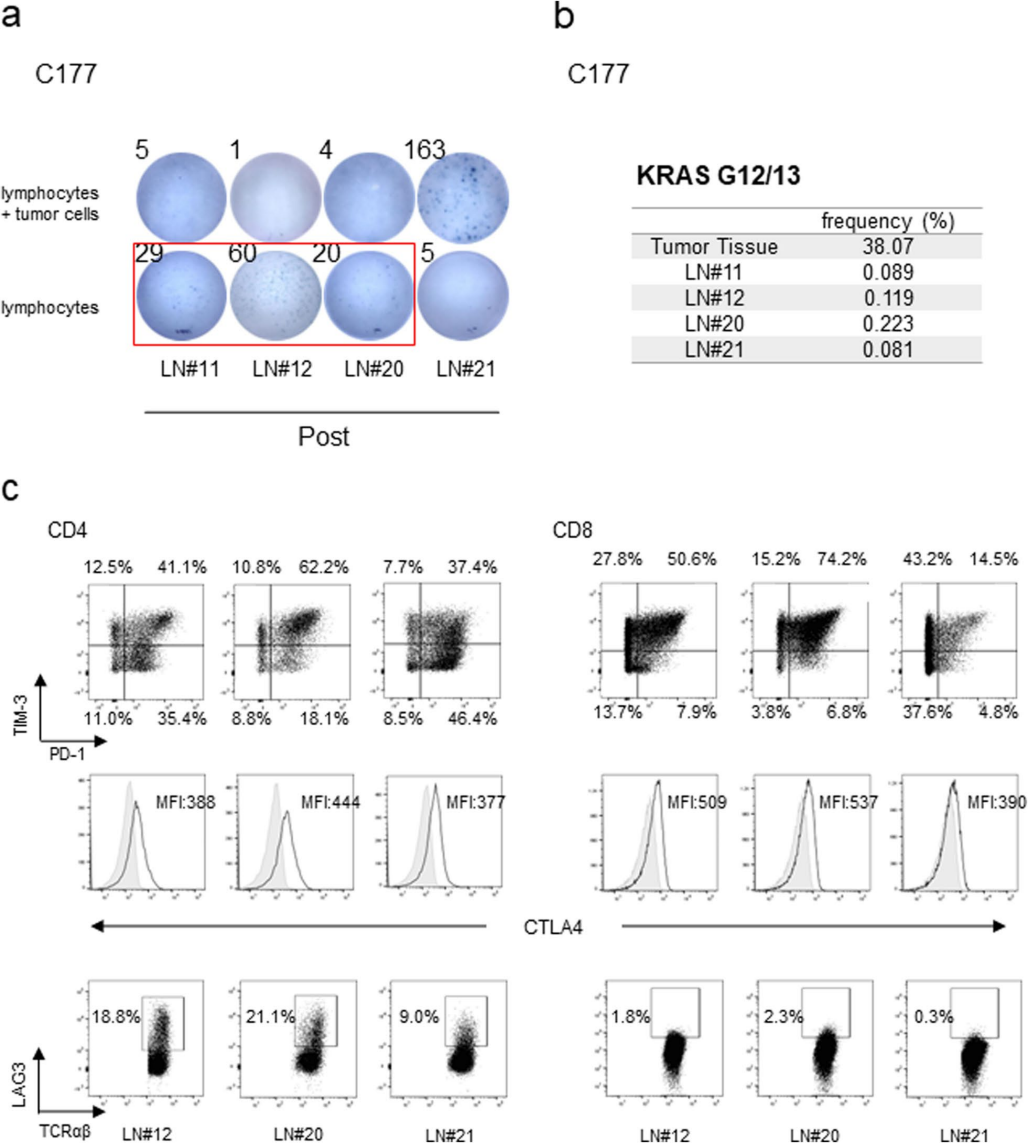
Inhibitory T cell expansion induced induction failure of cytotoxic CD8 T cells. **a** Lymphocytes co-culture with autologous tumor-cells for 2 weeks were rested without tumor cells for 2 days. Subsequently, lymphocytes (1 × 10^5^/well) were stimulated by autologous tumor-cells (1 × 10^5^/well) for 24 h and IFNγ secretions were detected by ELISPOT assay. Only lymphocytes samples were negative control. Experiments were conducted in duplicate. **b** Evaluation of KRAS mutation status on codon G12/G13 by ddPCR by using gDNA of tumor tissues and lymph nodes of C177. **c** Lymphocytes were stained with anti-TCRαβ, CD4, CD8α, TIM-3, PD-1, CTLA4 and LAG3 antibodies. TIM-3, PD-1, CTLA4 and LAG3 expression on CD4 or CD8 T cells were evaluated by flow cytometry respectively. The number indicates the proportion and mean fluorescence intensity (MFI) of each population in live cells

To investigate the factor(s) that might cause differences in anti-tumor reactivities, we focused on case C177 because lymphocytes from four lymph nodes showed different reactivities in IFNγ production. We defined “positive in IFNγ ELISPOT assays” when the number of positive spots became more than 1.5 folds higher than the corresponding negative controls. Although lymphocytes from LN#21 of this patient showed high IFNγ production after the co-culture with autologous tumor cells, those from the remaining three lymph nodes (LN#11, 12 and 20) did not show any positive signs (Fig. 1a). Interestingly, these three lymph nodes showed high IFNγ secretion in no stimulation condition with tumor cells (as negative controls) at the same experiment (Fig. 2a and Additional file 2: Fig. S2a). We cultured lymphocytes to expand tumor-reactive T cells with tumor cells at a 20:1 ratio (lymphocytes: cancer cells, respectively) in the presence of anti-PD1 antibody. Then after 2 days of culture of lymphocytes without tumor cells or anti-PD1 antibody, we stimulated lymphocytes with tumor cells at the 1:1 ratio and found that IFNγ secretion was reduced by the addition of tumor cells, implying IFNγ production in lymphocytes of LN#11, 12 and 20 were likely to be inhibited by some factors from the tumor cells. Although these four lymph nodes were pathologically diagnosed to be non-metastatic, we detected the *KRAS* mutation, which was found in the primary tumor, in DNA isolated from all four lymph nodes with ddPCR, suggesting a presence of a small number of tumor cells or phagocytosed tumor cells that might have primed anti-tumor reactivity (Fig. 2b).

We then investigated expression levels of four immuno-inhibitory receptors (PD-1, TIM3, CTLA-4 and LAG3) in lymphocytes of LN#12, 20, and 21 (we were unable to examine LN#11 because of a very small number of lymphocytes obtained). TIM3- and PD-1-positive cells among CD8^+^ T populations of LN#12 and 20 were higher than in those of LN#21 and LN#23, LN#26 and LN#28 of C207 which showed tumor-reactivity (Fig. 2c and Additional file 2: Fig. S2b). The proportion of TIM3 expressing CD8^+^ T cells in lymphocytes after co-culture with autologous tumor cells were lesser than that of TILs (Additional file 2: Fig. S2b and S2c). These results indicate that tumor-reactive lymphocytes after co-culture were less exhausted phenotype than TILs [31, 32]. Moreover, the CTLA-4 expression level was lower in CD8^+^ populations of LN#21 than those of LN#12 and LN#20. Furthermore, the LAG3 expression level was lower in both CD4^+^ and CD8^+^ populations of LN#21 compared with those in LN#12 and LN#20 (Fig. 2c). These results indicate that CD4^+^ and CD8^+^ T cells expressing inhibitory receptor molecules dominantly increased in LN#12 and LN#20, but the number of such cells was low in LN#21. Then, we further investigated the transcript profiling of lymphocytes before and after the co-culture with tumor cells. Lymphocytes of LN#21 showed distinct RNA expression pattern from three other lymph nodes after the co-culture with tumor cells (Additional file 3: Fig. S3a). Gene set enrichment analysis showed elevated expressions of genes involved in a KEGG_systemic_Lupus_erythematosus pathway (CD80, CD86 and HLA class II molecules) although the difference was not statistically significant.

Since the HLA class II-CD4 pathway may affect the differences in T cell responses, we examined HLA class II expression on tumor cells (Additional file 3: Fig S3b). C215 sigmoid colon did not express HLA class II and which tumor cells could not induce tumor reactive CD8^+^ T cells effectively. Therefore, we investigated CD4 phenotypes before and after the co-culture with tumor cells. Because *TBX21* is known as a master transcriptional regulator of Th1 and *GATA3* as that of Th2 [33], we examined relative *TBX21/GATA3* RNA expression ratio by real-time PCR. All samples (except C215), which showed the increase of the *TBX21/GATA3* ratio after the co-culture with tumor cells, revealed the increase of the anti-tumor reactivity (Additional file 4: Fig. S4a). Moreover, the addition of IL-12 and anti-IL-4 antibody in this culture system (Th1 induction) increased Tbx21 expression (Additional file 4: Fig. S4b) and reinforced cytotoxic activity of lymphocytes in C215, which was measured by a Perforin ELISPOT assay (Additional file 4: Fig. S4c). These results suggest that antigen presentation via HLA class II molecules and CD4 phenotypes may affect anti-tumor CTL induction in our culturing system.

### T cells clonotypes in lymph nodes shared with those in TILs proliferated by co-culture with tumor cells

**Fig. 3:**
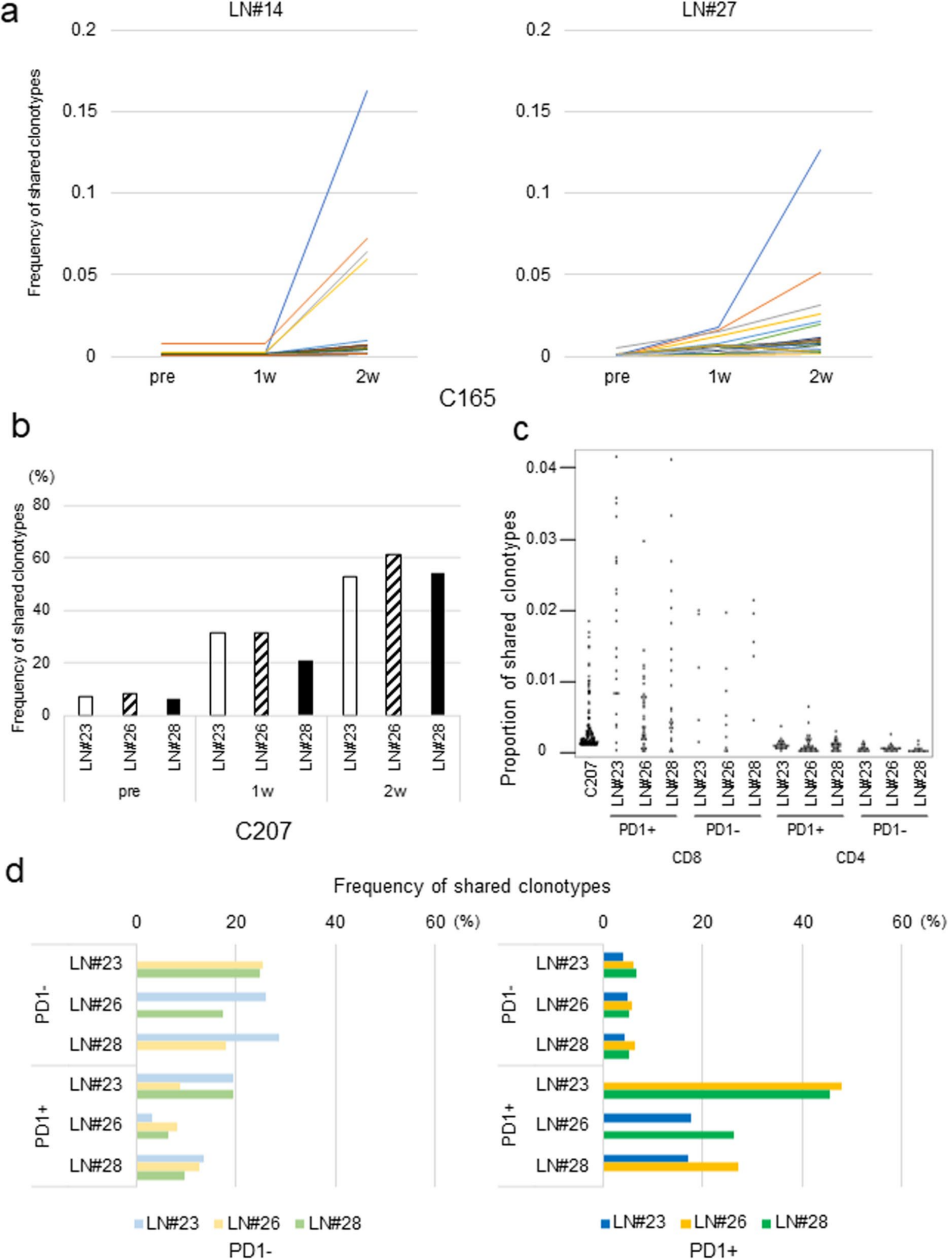
Shared clonotypes with TILs were expanded by co-culture with tumor cells. **a** TCRβ clonotypes of lymphocytes before, 1 and 2 weeks after co-cultured with tumor-cells were compared with those of 4 TIL fragments which were recognized over 0.1% in at least 1 TIL fragment of C165. The number indicates the % of the frequency of shared clonotypes detected in each lymph node. **b** TCR clonotypes of lymphocytes before and after co-cultured with tumor-cells were compared with those of TILs which occupied over 0.01% in total T cells of C207. The number indicates the % of the frequency of shared clonotypes detected in each lymph node. **c** Lymphocytes of non-metastatic lymph nodes of C207 were stained with anti-TCRαβ, CD4, CD8α and PD-1 antibodies. CD4 and CD8 T cells were sorted by PD1 expression pattern. TCRβ clonotypes of TILs were compared with each population in each lymph nodes. The number indicates the frequency of shared clones. **d** TCRβ clonotypes of lymphocytes that occupied more than 0.1% of CD8 T cells in each lymph nodes were compared between lymph nodes by PD1 expression pattern. The number indicates the % of the frequency of shared clonotypes between each sample

**Fig. 4:**
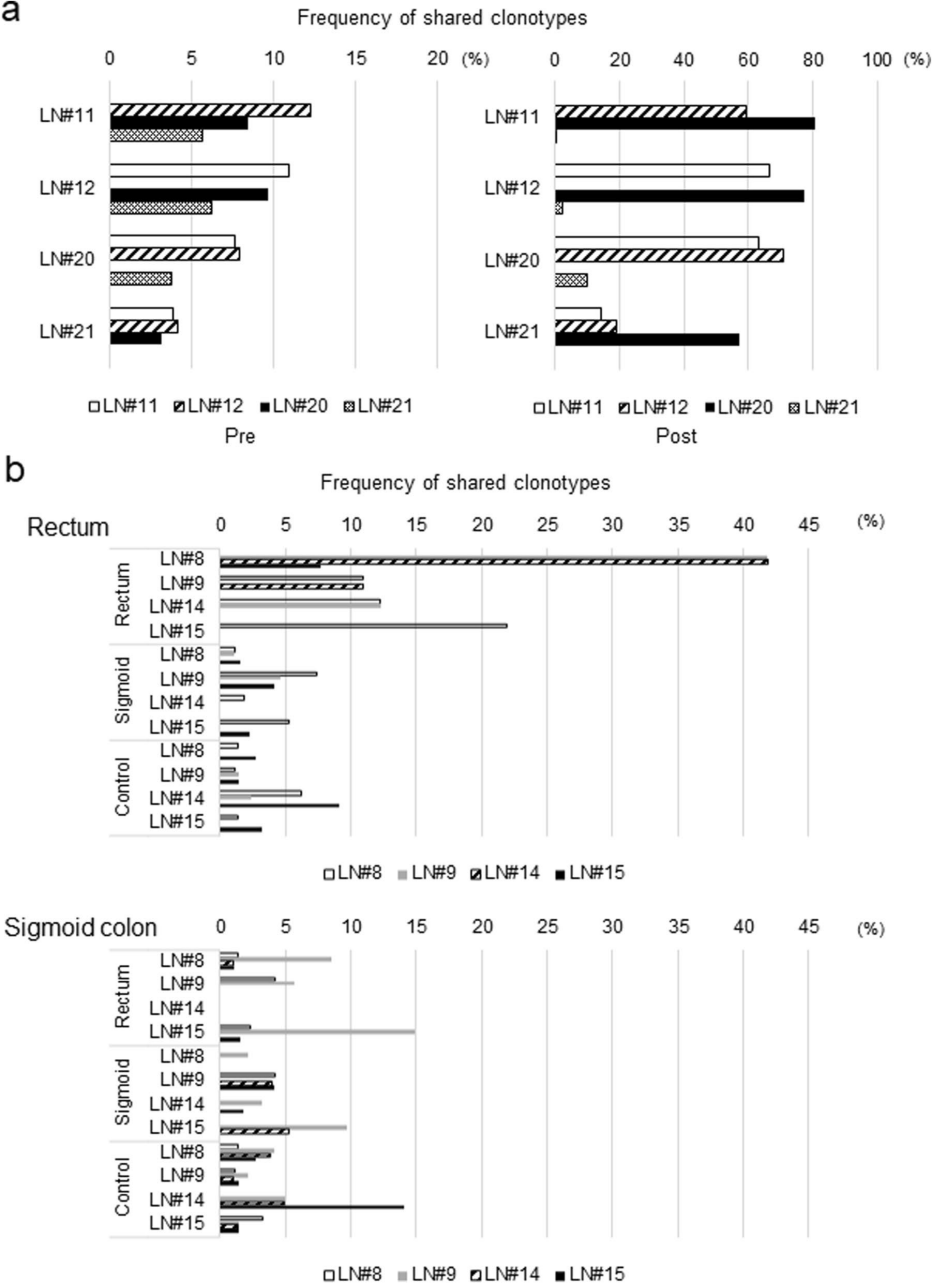
Tumor reactivities depend on shared TCR clonotypes between lymph nodes. **a**, **b** TCRβ clonotypes of lymphocytes that occupied more than 0.1% of T cells in lymph node were compared between each lymph node before and after co-culture with tumor-cells. The number indicates the % of the frequency of shared clonotypes in each lymph node. C177 (**a**) and C215 (**b**). Rectum and sigmoid colon show the condition with respective tumor cell lines for co-culture and control without tumor cell lines for co-culture period

TILs have been considered as clinically good sources of tumor-reactive T cells although it is now well known that TILs are functionally impaired in many cases [34]. We compared TCRβ clonotypes of TILs isolated from 4 different portions of primary tumor tissue with those of lymphocytes from two lymph nodes (#14 and #27) cultured with autologous tumor cells in case C165. T cells of #14 and #27, which shared TCRβ clonotypes with TILs cultured from four portions, were expanded significantly (Fig. 3a). In addition, we compared TCRβ clonotypes of lymphocytes in lymph nodes with those of tumor-infiltrating lymphocytes after single cell suspension of tumor tissues in case C207. Similarly, T cells from the two lymph nodes of C165, which shared clonotypes with TILs in a primary tumor, expanded by the co-culture with tumor cells (Fig. 3b). Then, to examine the type(s) of T cell clones that were expanded by co-culture with tumor cells, we sorted PD-1-expressing (or not expressing) CD4^+^ or CD8^+^ T cells from non-metastatic lymph nodes and compared their clonotypes with those of TILs in case C207. T cell receptor clonotypes of PD-1-expressing CD8^+^ T cells in lymph nodes shared more commonly with those of TIL than those of PD-1-negative cells (Fig. 3c). Furthermore, we compared clonotype sharing of CD8^+^ T cells according to PD-1 expression levels of three lymph nodes and found that the commonality of clonotypes with TILs was correlated with the PD-1 expression pattern of each lymph node (Fig. 3d). These data suggest that some CD8^+^ T cell clones having TCRs commonly with TILs might be activated in these three lymph nodes in vivo as indicated by PD-1 expression. Furthermore, we compared TCR clonotypes of four lymph nodes in case C177. Among lymphocytes separated from four lymph nodes, one sample from LN#21 showed the tumor-reactivity measured by an IFNγ ELISPOT assay after the co-culture with tumor-cells. TCRβ clonotypes in LN#21 indicated no similarity with those of the remaining three lymph nodes, LN#11, #12 and #20 (Fig. 4a) while TCR clonotypes of lymphocytes in LN#11, #12 and #20 showed the similarities with each other after the co-culture with tumor cells.

Similar results were obtained for four lymph nodes of a case C215, who had cancers in the sigmoid colon and rectum simultaneously. Lymphocytes in 3 (#8, #9 and #14) of 4 lymph nodes co-cultured with rectal tumor cells showed positive results in an IFNγ ELISPOT assay. On the other hand, lymphocytes in LN#9 were IFNγ-positive when they were co-cultured with sigmoid tumor cells while lymphocytes in the remaining lymph nodes did not show any immune-reactivity against the sigmoid colon tumor cells. We found higher numbers of common TCR clonotypes among lymphocytes cultured with the rectal tumor than those co-cultured with the sigmoid tumor (Fig. 4b). These results suggest that TCR commonality between lymphocytes in lymph nodes after the co-culture with tumor-cells and those of tumor tissue may affect tumor-reactivity of lymph nodes. Interestingly, LN#8 and LN#9 belonged to a D2 region of the sigmoid tumor and were not regional lymph nodes of the rectal tumor. Although the lymphatic flow in this patient was not exactly known, our results indicate that lymph nodes (LN#8 and LN#9) relatively far from a primary tumor might be also applicable for the ACT when TAA-reactive T cell clones could be expanded by this approach.

## Discussion

TDLNs, where anti-tumor immune activity is primarily induced, play pivotal roles to effectively generate anti-tumor T cell responses [18, 19, 35]. TDLNs are usually excised during surgery along with a primary tumor(s) to remove possible residual tumor cells and are examined for diagnosis with/without metastatic tumor cells in these lymph nodes [36]. It is now well-known that extensive dissection of LNs provided little clinical benefit on the long-term survival for MSI-H/dMMR CRC patients [37]. It is also suspected that the preservation of lymph nodes is likely to be beneficial for host immune responses against cancer cells [38]. In this study, we explored the possibility of the use of TDLNs for the ACT and have shown the presence of tumor-reactive T cells in non-metastatic lymph nodes as well as a possibility for expansion of tumor-reactive cytotoxic T cells in vitro by co-culture with autologous tumor cells. As expected, tumor-reactive T cells in lymph nodes in Lynch syndrome (C207) were already activated and proliferated in vivo, lymphocytes needed co-culture with autologous tumor-cells in other samples. The advantage of ACT using TILs is obtaining a large number of T cells that may have anti-tumor activity. The establishment of TILs for ACT usually needs two phases [39]. In the first phase, lymphocytes were separated from tumor tissues and were cultured in vitro, and then were activated in the medium containing a high concentration of IL-2 to further increase tumor-reactive T cell clones. In the second phase, lymphocytes are further expanded up to 1000 folds (often up to 10^11^ cells) in rapid expansion procedure condition [7, 40]. However, this approach did not work well for advanced-stage CRC patients [5, 41], but it has been investigated by the combination with immune checkpoint inhibitors [42]. Exhausted phenotype of CD8^+^ T cells were thought to be related to impairment of cytotoxicity [34]. In fact, TILs showed more exhausted phenotype than lymphocytes after co-culture with autologous tumor cells. On the other hand, it was shown that a smaller number of lymphocytes (median 153 × 10^6^ cells) might work effectively for stage IV colorectal cancer patients when lymphocytes were isolated from sentinel lymph nodes [21]. In any case, the number of tumor-reactive T cells should certainly be one of the key factors for the success of adoptive cell therapy. In our experiences, it is not so difficult to obtain 1 × 10^7^ or more lymphocytes from one non-metastatic lymph node, which can be expanded to several times by the 2-week culture with autologous tumor cells. Furthermore, if we combine the rapid expansion procedure in our culture condition, it would be possible to obtain a much larger number of tumor-reactive T cells.

In many cases, cell therapy has been provided to advanced-stage patients who had metastatic, refractory or relapse tumors. In this study, we established autologous tumor-cell lines from fresh tumor tissues. It would be difficult to prepare autologous tumor cell lines in advance for all patients in clinical study. We will get tumor-cell lines more reliably even from cryopreserved tumor tissues if we use organoid culture system [43]. Moreover, lymphocytes of TDLNs can be kept for a long time after single cell suspension and these cells can be activated, expanded and good cell sources for cell therapy whenever patients experience relapse.

Our data have indicated that levels of anti-tumor-reactivity of lymphocytes after the co-culture with tumor cells might be predicted by the numbers and frequencies of expanded lymphocytes that shared clonotypes with TILs in primary sites. In this study, we were unable to examine the type of antigens that were recognized by lymphocytes proliferated by co-culture with autologous tumor cell lines. We could not also confirm the specificity to tumor cells, but since lymphocytes recognizing self-antigens (except some cancer-testis antigens) are usually eliminated, we assume most or at least some of expanded lymphocytes recognize tumor-specific antigens. Although lymphocytes cultured from lymph nodes would be safe as TILs [24], it is necessary to check the cross-reactivity with normal cells when applying our approach in the clinic to make sure the safety of the patients. Furthermore, despite we would not be able define the types of lymph node that might have a higher potential as good cell sources for the ACT before the co-culture with autologous tumor cells because we found no meaningful difference in the cell population levels, cell phenotypes or RNA expression profiles. However, we might be able to do this by characterization of T cell populations and phenotypes after the co-culture with tumor cells [42]. In addition, T cell persistency and proliferation capability in vivo after the cell transfusion seemed to be crucial for the clinical effectiveness of ACT; a T cell stem-like phenotype is possibly related to the in vivo persistency [44, 45].

Notably, we could enrich tumor-reactive T cells from non-nearest lymph nodes of multiple patients; these lymph nodes did not belong to the nearest regional lymph node group of the tumor. Regional lymph nodes for CRC are categorized as D1, D2 and D3 by anatomical locations. D1 is the nearest and D3 is relatively distant from the primary site. LN#8 and LN#9 of C215 (regional lymph nodes of sigmoid colon tumor of this patient) were located anatomically far from the primary rectal tumor and were not regional lymph nodes of this rectal cancer, but lymphocytes from these lymph nodes showed the immune reactivity against rectal tumor cells. Since the flow of lymphatic vessels is complicated, it is not conclusive that non-metastatic lymph nodes located relatively far from the primary site have been activated by TAAs in vivo. We assume that all lymph nodes, which contain tumor-reactive T cells even at a very low level, can be used as cell sources for ACT therapy when lymphocytes are cultured with autologous tumor cells.

In summary, our findings imply that non-metastatic lymph nodes might be applicable as cell sources for adoptive cell therapy. These data highlighted the utility of non-metastatic lymph nodes for a new therapeutic option for CRC patients with poor prognosis.

## Supplementary Information

**Additional file 1:** **Figure S1.** Screening and expansion of tumor-reactive T cells in lymph nodes. **a** Lymphocytes (1×105/well) cultured from non-metastatic and metastatic lymph nodes were stimulated by autologous tumor-cell lines (1×105/well) for 24 hours and IFNγ secretions were detected by ELISPOT assay. Only lymphocytes samples were negative control and lymphocytes + anti-CD3 antibody (1μg/well) samples were positive control. A sample was considered positive when the spot number is more 1.5 folds than the negative control. Experiments were conducted in duplicate. **b** Scheme of enrichment of tumor-reactive T cells in vitro.

**Additional file 2: Figure S2.** Screening of tumor-reactive T cells and TIM-3 and PD-1 expression on CD8+ T cells. **a** Second results of screening tumor-reactive T cells (Fig.S1a). **b** Lymphocytes were stained with anti-TCRαβ, CD4, CD8α, TIM-3 and PD-1 antibodies. TIM-3 and PD-1 expression on CD8 T cells were evaluated by flow cytometry. **c** The proportion of TIM-3 expressing CD8+ T cells cultured from tumor tissues (n=3 C149, n=3 C152).)

**Additional file 3: Figure S3. **HLA Class II expression was related to induction of tumor-reactive T cells. **a** Heatmap displaying top 100 expressing genes of LN#21 after co-culture which were over 4 folds higher expression than LN#11, 12 and 20. **b** Tumor cell lines were stimulated by IFNγ(200IU/ml) for 24 hors and HLA class II expression were evaluated by flow cytometry.

**Additional file 4: Figure S4.** Th1 phenotype reinforces cytotoxic activity. **a** Tbx21 and GATA3 mRNA expression in T cells was quantified by real-time polymerase chain reaction using TaqMan gene expression assays. Expression levels were normalized to Actin expression. The relative expression of Tbx21 to GATA3 was considered to be the balance of the Th1/Th2 phenotype. **B** Lymphocytes of LN#9 and LN#14 in C215 were cultured with rectum tumor or sigmoid colon tumor cell lines for 2 weeks in Th1 induction (IL-12 10ng/ml, anti-IL4 antibody 10μg/ml) or not (control). These two conditions contained IL2 (200 IU/ml), anti-PD1 antibody (10μg/ml) and anti-CD28 antibody (5μg/ml) same as the previous experiment. Lymphocytes were collected and Tbx21 and GATA3 mRNA expression in T cells was quantified by real-time polymerase chain reaction using TaqMan gene expression assay. **c** Lymphocytes were cultured with autologous tumor-cells for 2 weeks with or without Th1 induction. Subsequently, lymphocytes were cultured in only lymphocytes medium for 2 days and lymphocytes were stimulated with tumor cell lines (1×105/well) for 24 hours again and Perforin secretions were detected by ELISPOT assay. Experiments were conducted in duplicate.

**Additional file 5: Table S1.** Genomic Characteristics of tumor cells of C215

## Data Availability

The data generated and analyzed during the current study are available from the corresponding author upon a reasonable request.

## Abbreviations

ACT: Adoptive cell therapy
CRC: Colorectal cancer
CTL: Cytotoxic T lymphocyte
TAA: Tumor-associated antigen
TCR: T cell receptor
TDLNs: Tumor-draining lymph nodes
TILs: Tumor-infiltrating lymphocytes
MMR: Mismatch repair
MSI: Microsatellite instability
