# Supplementary figures and images for: Lymphocytes in tumor-draining lymph nodes co-cultured with autologous tumor cells for adoptive cell therapy
Source: J Transl Med. 2022 May 23;20:241. doi: 10.1186/s12967-022-03444-1 (PMC9125345; doi:10.1186/s12967-022-03444-1)

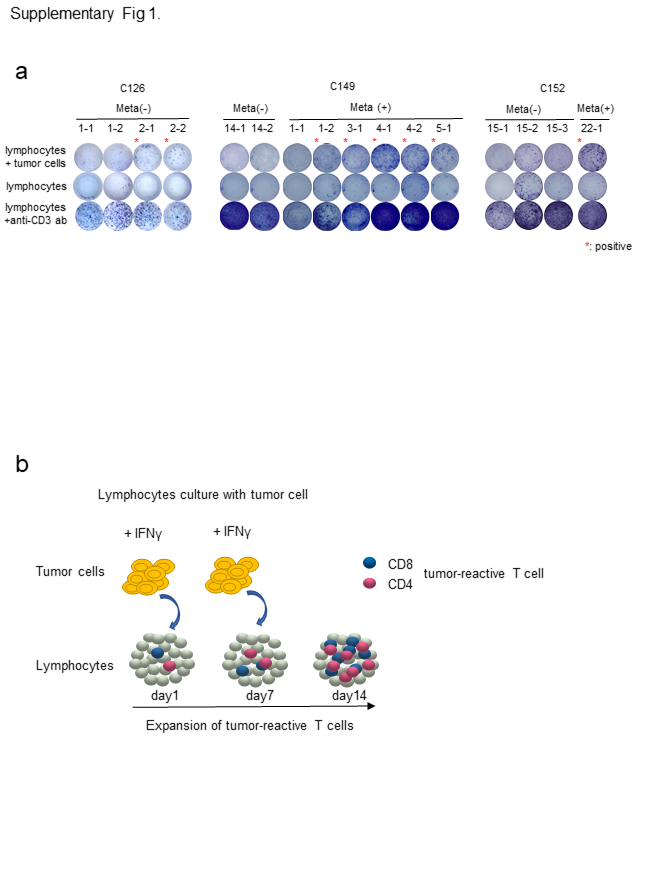

Supplement: Supplementary file 1 — Additional file 1: Figure S1. Screening and expansion of tumor-reactive T cells in lymph nodes. a Lymphocytes (1×10^5^/well) cultured from non-metastatic and metastatic lymph nodes were stimulated by autologous tumor-cell lines (1×10^5^/well) for 24 hours and IFNγ secretions were detected by ELISPOT assay. Only lymphocytes samples were negative control and lymphocytes + anti-CD3 antibody (1μg/well) samples were positive control. A sample was considered positive when the spot number is more 1.5 folds than the negative control. Experiments were conducted in duplicate. b Scheme of enrichment of tumor-reactive T cells in vitro. [file 12967_2022_3444_MOESM1_ESM.tif]

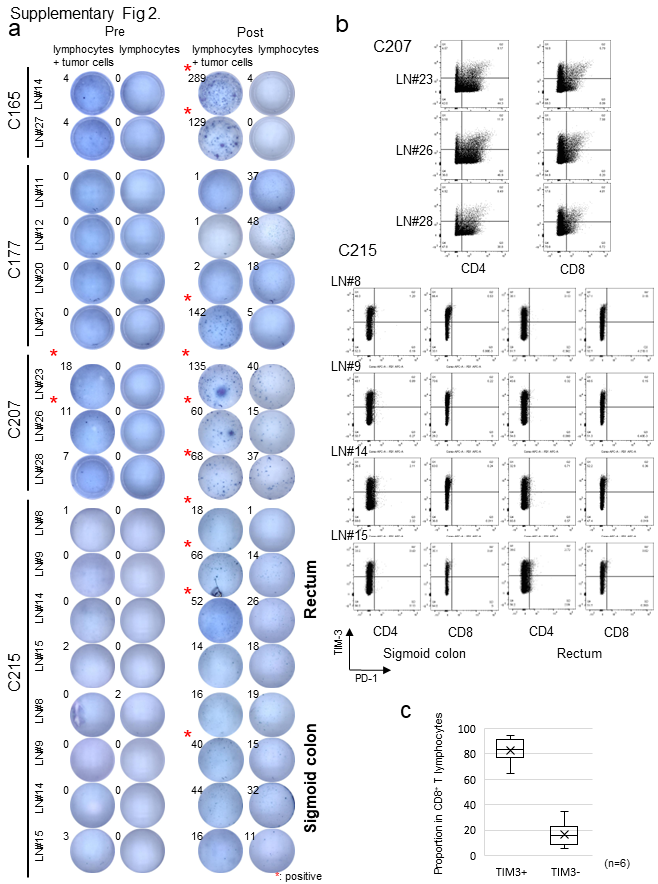

Supplement: Supplementary file 2 — Additional file 2: Figure S2. Screening of tumor-reactive T cells and TIM-3 and PD-1 expression on CD8+ T cells. a Second results of screening tumor-reactive T cells (Fig.S1a). b Lymphocytes were stained with anti-TCRαβ, CD4, CD8α, TIM-3 and PD-1 antibodies. TIM-3 and PD-1 expression on CD8 T cells were evaluated by flow cytometry. c The proportion of TIM-3 expressing CD8+ T cells cultured from tumor tissues (n=3 C149, n=3 C152).) [file 12967_2022_3444_MOESM2_ESM.tif]

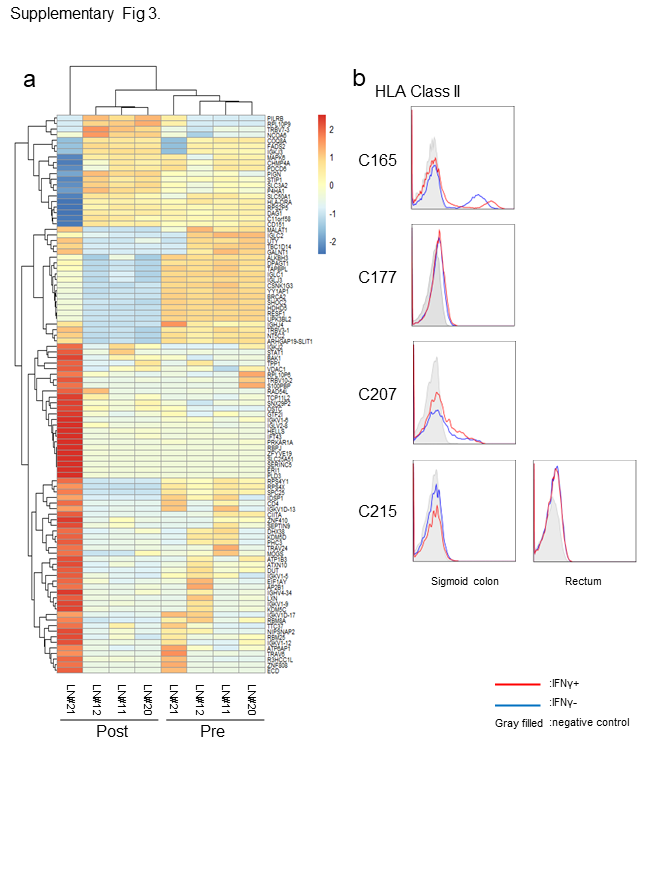

Supplement: Supplementary file 3 — Additional file 3: Figure S3. HLA Class II expression was related to induction of tumor-reactive T cells. a Heatmap displaying top 100 expressing genes of LN#21 after co-culture which were over 4 folds higher expression than LN#11, 12 and 20. b Tumor cell lines were stimulated by IFNγ(200IU/ml) for 24 hors and HLA class II expression were evaluated by flow cytometry. [file 12967_2022_3444_MOESM3_ESM.tif]

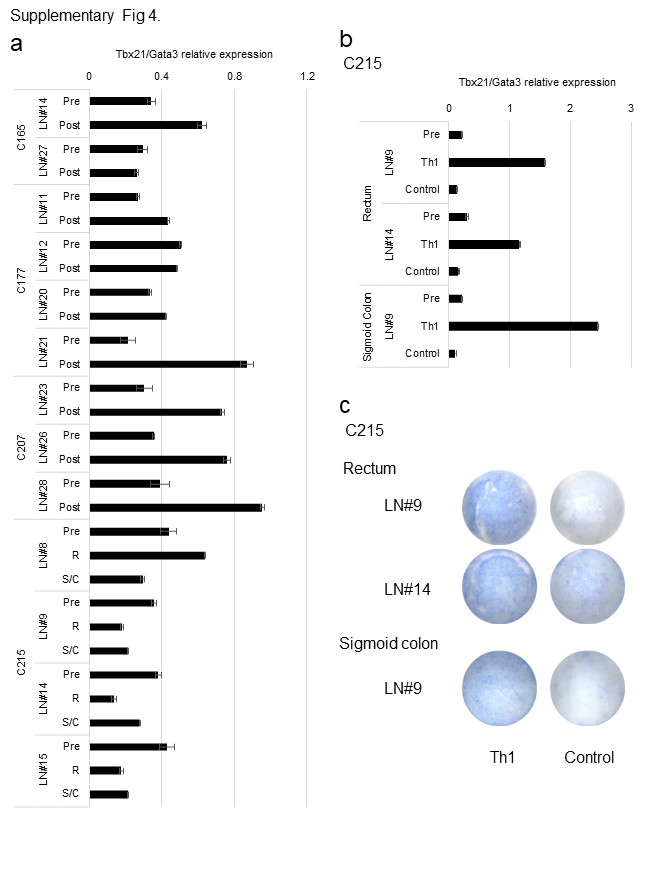

Supplement: Supplementary file 4 — Additional file 4: Figure S4. Th1 phenotype reinforces cytotoxic activity. a Tbx21 and GATA3 mRNA expression in T cells was quantified by real-time polymerase chain reaction using TaqMan gene expression assays. Expression levels were normalized to Actin expression. The relative expression of Tbx21 to GATA3 was considered to be the balance of the Th1/Th2 phenotype. B Lymphocytes of LN#9 and LN#14 in C215 were cultured with rectum tumor or sigmoid colon tumor cell lines for 2 weeks in Th1 induction (IL-12 10ng/ml, anti-IL4 antibody 10μg/ml) or not (control). These two conditions contained IL2 (200 IU/ml), anti-PD1 antibody (10μg/ml) and anti-CD28 antibody (5μg/ml) same as the previous experiment. Lymphocytes were collected and Tbx21 and GATA3 mRNA expression in T cells was quantified by real-time polymerase chain reaction using TaqMan gene expression assay. c Lymphocytes were cultured with autologous tumor-cells for 2 weeks with or without Th1 induction. Subsequently, lymphocytes were cultured in only lymphocytes medium for 2 days and lymphocytes were stimulated with tumor cell lines (1×10^5^/well) for 24 hours again and Perforin secretions were detected by ELISPOT assay. Experiments were conducted in duplicate. [file 12967_2022_3444_MOESM4_ESM.tif]
