# Supplementary material for: Lymphocytes in tumor-draining lymph nodes co-cultured with autologous tumor cells for adoptive cell therapy
Source: J Transl Med. 2022 May 23;20:241. doi: 10.1186/s12967-022-03444-1 (PMC9125345; doi:10.1186/s12967-022-03444-1)
Supplement: Supplementary file 5 — Additional file 5: Table S1. Genomic Characteristics of tumor cells of C215 [file 12967_2022_3444_MOESM5_ESM.docx]

Supplementary Table.1 Genomic Characteristics of tumor cells of C215

| Rectum tumor | | | |  | Sigmoid colon tumor | | | |
| --- | --- | --- | --- | --- | --- | --- | --- | --- |
| Gene | type | protein | frequency (%) |  | Gene | type | protein | frequency (%) |
| APC | SNV | p.Arg216Ter | 84.04 |  | TCF7L2 | SNV | p.Arg471Cys | GT=3.72,TG=89.89 |
| MAP2K4 | INDEL | p.Ser393AlafsTer20 | 73.68 |  | FBXW7 | SNV | p.Ser582Leu | C=12.32,CA=41.23 |
| TP53 | SNV | p.Gly245Ser | 71.33 |  | TP53 | SNV | p.Arg273Pro | 82.51 |
| PTPRT | SNV | p.Arg1139Gln | 63.64 |  | CDH2 | SNV | p.Arg807Gln | 82.31 |
| TCF7L2 | SNV | p.Met1? | 47.27 |  | ARID1A | SNV | p.Tyr222Ter | 57.92 |
| ARID1A | SNV | p.Glu1803Ter | 44.26 |  | SYNE1 | SNV | p.Val1167Ile | 42.12 |
| NRAS\|CSDE1 | SNV | p.Gly12Asp\|p.? | 42.13 |  | CSMD3 | SNV | p.Leu3397Phe | 39.86 |
| ADGRL3 | SNV | p.Arg664His | 37.66 |  | ESR1 | SNV | p.Leu327Pro | 38.01 |
| ESR1 | SNV | p.Arg300Cys | 37.18 |  | CYLD | SNV | p.Gln134Lys | 27.42 |
| BLM | SNV | p.Gln1343Ter | 27.85 |  | IGF2\|IGF2-AS | SNV | p.Glu42Lys\| | 22.56 |
| PTPRT | SNV | p.Met775Lys | 23.47 |  | KIT | SNV | p.His263Gln | 9.42 |
| KMT2C | SNV | p.Asp348Asn | 8.05 |  | KMT2C | SNV | p.Pro350= | 8.95 |
| DCC | SNV | p.Gly1031Arg | 7.23 |  | KMT2C | SNV | p.Asp348Asn | 8.65 |
| TSHR | SNV | p.Thr513Pro | 6.67 |  | DCC | SNV | p.Gly1031Arg | 7.58 |
| SYNE1 | SNV | p.Thr1515Asn | 6.32 |  | CREBBP | SNV | p.Ser2382Phe | 6.84 |
| CSMD3 | SNV | p.Pro2965His | 5.37 |  | FGFR2 | SNV | p.Asn727Ser | 5.63 |
